# Essential Oil Microemulsions Inactivate Antibiotic-Resistant Bacteria on Iceberg Lettuce during 28-Day Storage at 4 °C

**DOI:** 10.3390/molecules27196699

**Published:** 2022-10-08

**Authors:** Stephanie Arellano, Libin Zhu, Govindaraj Dev Kumar, Bibiana Law, Mendel Friedman, Sadhana Ravishankar

**Affiliations:** 1 School of Animal and Comparative Biomedical Sciences, University of Arizona, 1117 E. Lowell Street, Tucson, AZ 85721, USA; 2 Center for Food Safety, University of Georgia, Griffin, GA 30223, USA; 3 USDA-ARS Western Regional Research Center, Albany, CA 94710, USA

**Keywords:** antimicrobials, microemulsions, Iceberg lettuce, *Escherichia coli* O157:H7, *Pseudomonas fluorescens*, oregano oil, cinnamon oil, lemongrass oil, shelf-life, food safety

## Abstract

The objective of this study was to investigate the antimicrobial activities of essential oil-based microemulsions in the wash water against *Escherichia coli* O157:H7 and *Pseudomonas fluorescens* on Iceberg lettuce. Evaluated wash microemulsions included oregano oil, lemongrass oil, and cinnamon oil, along with a plant-based emulsifier for improved solubility. Iceberg lettuce was inoculated for 2 min with *E. coli* O157:H7 (6.0 log CFU/g) or *P. fluorescens* (6.0 log CFU/g) and then dip-treated in a phosphate buffered saline (PBS) control, 50 ppm chlorine, 3% hydrogen peroxide treatment or a 0.1%, 0.3%, or 0.5% microemulsion solution. Treated leaves were stored at 4 °C, and analyzed for surviving bacteria on days 0, 3, 7, 10, 14, 21, and 28. Efficacies of the antimicrobials were concentration and storage-time dependent. There was a 1.26–4.86 log CFU/g reduction in *E. coli* O157:H7 and significant reductions (0.32–2.35 log CFU/g) in *P. fluorescens* during storage at days 0–28 (*p* < 0.05). The 0.1% oregano oil microemulsion resulted in the best visual appeal in Iceberg leaves inoculated with *E. coli* O157:H7 and showed better improvement in the quality of the Iceberg leaves inoculated with spoilage bacteria *P. fluorescens*. The results suggest that 0.5% cinnamon and 0.3% oregano oil treatments have the potential to provide natural, eco-friendly, and effective alternatives to chemicals for the decontamination of leafy greens, eliminating *E. coli* O157:H7 and *P. fluorescens*.

## 1. Introduction

The consumption of fresh-cut leafy greens such as Iceberg lettuce and cruciferous vegetables provides nutrients including vitamin C, B1, B2, and folate, all of which support the increased response of immune activation [1]. The shift towards healthier lifestyles has resulted in the incorporation of more fresh fruits and vegetables in peoples’ diets, but this has increased the burden of foodborne illness globally. Shiga toxin-producing *Escherichia coli* O157:H7 (STEC) was responsible for over half (56.5%) of the 23 produce-related multistate outbreaks that were due to pathogenic *E. coli* occurring in years 2010 to 2017. Additionally, this has created an immense financial loss of $254.8 million dollars annually in the United States [2]. More recently, *E. coli* O157:H7 associated multistate outbreaks of 2018 that implicated Romaine lettuce grown in Yuma, AZ, resulted in 240 case-patients, 104 hospitalizations, 28 developing hemolytic uremic syndrome, and 5 deaths [3]. 

The safety and quality of fresh fruits and vegetables rely on the industry practices at both pre- and post-harvest levels. Several factors may influence the spoilage of minimally processed vegetables which include the spoilage microbial population, initial visual quality of the perishable commodity, sanitizer used in the wash water, storage temperature, and packaging conditions. However, microbial spoilage is the most common cause of spoilage, leading to significant economic losses for the food industry. The spoilage organism *Pseudomonas fluorescens* has been widely known for its presence in minimally processed vegetables accounting for a majority of the bacterial spoilage in fresh-cut products [4]. Due to its psychotropic and pectinolytic nature, *P. fluorescens* can increase in numbers during storage at low temperatures and play an important role in the browning of minimally processed vegetables [5,6,7]. 

Minimally processed fruits and vegetables are highly susceptible to microbial proliferation because of their processing conditions that involve cutting, slicing, or peeling, allowing microbes to gain access to the nutritive substances found within the vascular tissue of the fruit or vegetable cut surfaces [8]. When the spoilage of minimally processed produce becomes apparent, the visual appeal that contributes to its freshness deteriorates and no longer presents an attractive convenience food item to consumers [9]. Various factors that may induce spoilage include the plants metabolic activity, pH, moisture content, and release of nutrients that cause senescence in vegetable tissue that may promote growth and survival of foodborne pathogens and spoilage bacteria [10]. Since these minimally processed vegetables are oftentimes consumed raw, intervention approaches such as natural, synthetic-free wash sanitizers and low-toxicity agents must be implemented to maintain the safety and quality of these products. 

Currently, established industry practices involve the use of chemical sanitizers such as chlorine that decontaminate fresh produce, resulting in 1–2 log reductions in foodborne pathogens, and limited reduction in spoilage microorganisms [11,12]. Treatment with organic acids [13,14] and with bacteriophages in combination with chlorine and mild heat also seem to be effective [15].

Consumers’ increasing concerns for chemical synthetic additives have prompted the further investigation of alternative methods for increasing food safety and quality in fresh-cut fruits and vegetables. Many of the plant essential oils have been widely recommended and classified as generally recognized as safe (GRAS) preservatives for food commodities based on their antimicrobial properties. Essential oils are volatile and comprise many compounds that provide antibacterial, antioxidant, antifungal, and antiviral properties. Naturally, essential oils have been known to control microorganisms related to food spoilage and thereby, extend the shelf-life of foods [16]. In addition, essential oils are natural additives that serve as promising antimicrobials, since they target a wide range of spoilage and pathogenic bacteria [17,18]. Yossa et al. [19] described the potential of natural plant-based compounds to inactivate *E. coli* O157:H7 on Iceberg lettuce during a 14-day period at 4 °C. The significant reductions exhibited by the cinnamaldehyde + Tween 20 treatment at various concentrations was in the range 2.5–2.89-log CFU/g during days 0–14 [19]. In addition, the effects of plant-based treatments were investigated against native microbiota on Iceberg lettuce and demonstrated to serve as moderators for spoilage bacteria throughout the 14-day storage period. This previous study also suggested the importance of seeking an emulsifying agent that will further increase solubility of the antimicrobial compounds [19]. In the present study, a modification to the natural plant-based antimicrobial treatments was investigated to determine if their efficacy would be improved with an emulsifier when compared to plant-based treatments without the emulsifier agent. 

The increased demand for minimally processed salad vegetables suggests that there is a need for better methods of preventing contamination especially during packaging or processing practices. To further investigate the potential uses of essential oil treatments against the foodborne pathogen *Escherichia coli* O157:H7 and spoilage organism *Pseudomonas fluorescens* on Iceberg lettuce, the present study evaluated the effectiveness of oregano, cinnamon, and lemongrass oil microemulsion treatments during a storage period of 28 days at 4 °C. See Friedman et al. [20] for a review of antimicrobial effects of plant essential oils and oil compounds and Nowotarska et al. [21] for suggested mechanisms that might govern their antimicrobial activities.

## 2. Results

**Figure 1:**
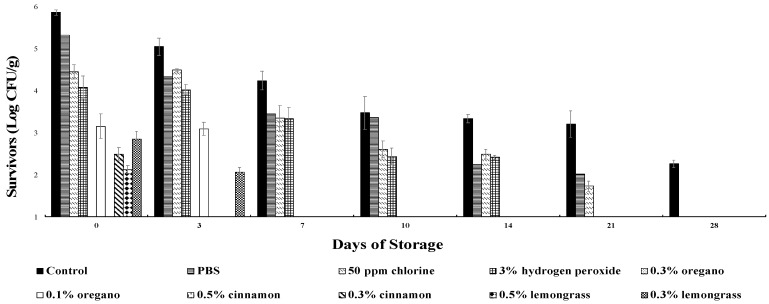
Survival of *E. coli* O157:H7 on Iceberg lettuce treated with 0.3 and 0.1% oregano; 0.5 and 0.3% cinnamon; and 0.5 and 0.3% lemongrass oil microemulsions for 2 min and stored at 4 °C for 28 days. All values are an average of three repeats. Error bars represent the standard deviation from the mean value.

Dose-dependent reductions in *E. coli* O157:H7 treated with oregano, cinnamon, and lemongrass oil microemulsions were observed as compared to controls. The initial *E. coli* O157:H7 population on inoculated Iceberg lettuce leaves was 5.86 log CFU/g (Figure 1). Treatment with 0.3% oregano and 0.5% cinnamon oil microemulsions showed a bactericidal effect during days 0–28, and the surviving population of bacteria on the leaves were below detection limit (1 log CFU/g). In contrast, the 50 ppm chlorine treatment resulted in a 0.56–1.48 log CFU/g reduction on days 0–21, and thereafter demonstrated no detection of survivors on day 28 (Figure 1). Treatments with 0.5% lemongrass and 0.3% cinnamon oil significantly reduced *E. coli* O157:H7 populations (3.74 and 3.36 log CFU/g, respectively) on Iceberg lettuce immediately after treatment, and had no surviving populations by day 3 (*p* < 0.05) in comparison to the 3% hydrogen peroxide that showed a 1.78 log CFU/g reduction, and the PBS control that showed a 0.54 log CFU/g reduction at day 0 (*p* < 0.05). No survivors in *E. coli* O157:H7 populations were observed on day 7 in lettuce treated with 0.3% lemongrass and 0.1% oregano oil (Figure 1). The *E. coli* O157:H7 populations were undetectable (<1 log CFU/g) in all the microemulsion treatments after 7 days of storage (Figure 1). 

### 2.1. Visual Inspection of E. coli O157:H7 Inoculated Iceberg Lettuce Leaves Treated with Essential Oil Microemulsions during 28 Days of Storage

**Figure 2:**
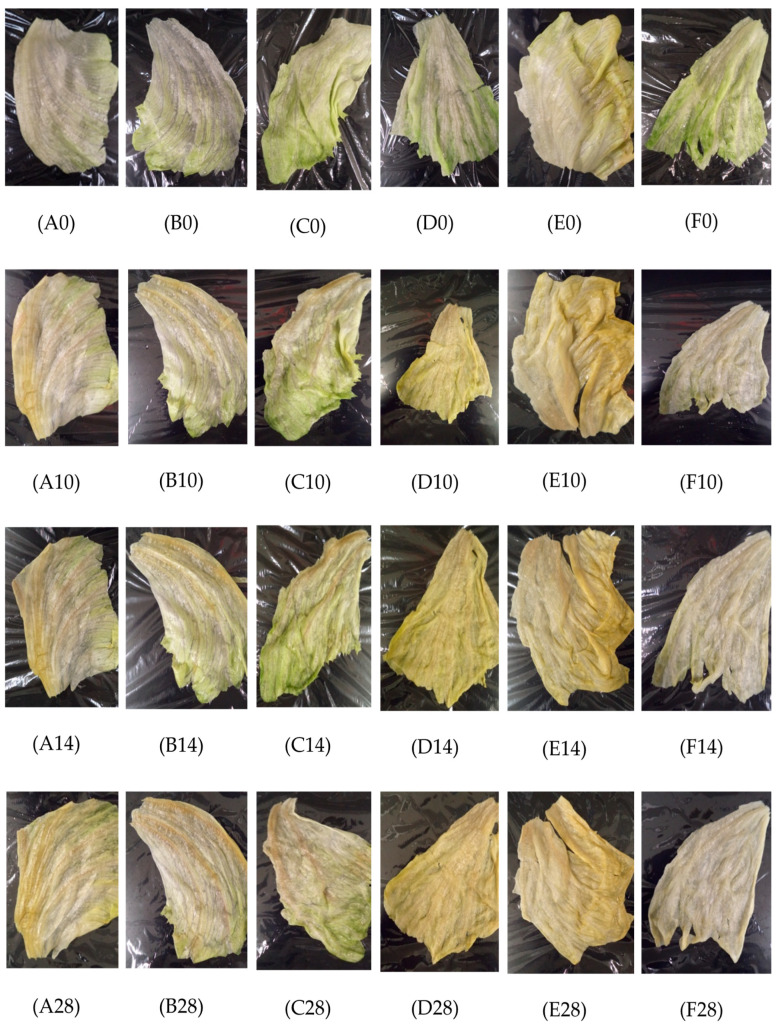
Visual observation of Iceberg lettuce leaves inoculated with *E. coli* O157:H7, and treated with 50 ppm chlorine, 3% hydrogen peroxide, and plant-based microemulsions and stored at 4 °C for 28 days. Letters denoted represent positive control (A0) on day 0, (A10) on day 10, (A14) on day 14, and (A28) on day 28; 50 ppm chlorine (B0) on day 0, (B10) on day 10, (B14) on day 14, and (B28) on day 28; 3% hydrogen peroxide (C0) on day 0, (C10) on day 10, (C14) on day 14, and (C28) on day 28; 0.3% oregano oil microemulsion (D0) on day 0, (D10) on day 10, (D14) on day 14, and (D28) on day 28; 0.5% cinnamon oil microemulsion (E0) on day 0, (E10) on day 10, (E14) on day 14, and (E28) on day 28; 0.5% lemongrass oil microemulsion (F0) on day 0, (F10) on day 10, (F14) on day 14, and (F28) on day 28.

The positive control leaves showed no signs of wilting or minimal browning, as they remained viable and kept their turgidity throughout the 28-day storage (Figure 2). The PBS control leaves showed slight discoloration on day 0, showing leaf intactness. There was a loss of moisture and minimal browning at the cut edges on days 7 and 10. On days 21 and 28, there were apparent signs of wilt, and browning located at the basal region of the leaf (Table 1). The 50 ppm chlorine-treated leaves kept their crisp texture but resulted in distinctive yellow discoloration on days 10, 14, and 28 with visible browning at the cut edges (Figure 2). The leaves treated with 3% hydrogen peroxide demonstrated signs of wilting on day 7, browning on days 14 and 21, and complete disintegration of the leaf on day 28, but they retained their greenness throughout the surface (Table 1). The leaves treated with 0.3% oregano oil remained viable, with slight olive-toned discoloration and loss of moisture during days 10, 14, and 28 (Figure 2). The leaves treated with 0.1% oregano oil showed no discoloration, retaining greenness across the leaf and a slight loss of moisture on days 0–28 (Table 1). For the leaves treated with 0.5% cinnamon oil, there was no discoloration shown, so the greenness remained visible. However, by day 28, slight yellowing and browning on the cut edge of the leaf along with loss of moisture were observed (Figure 2). The 0.3% cinnamon oil-treated leaves showed minimal olive-toned discoloration starting on day 10 and continuing through to day 28 (Table 1). Both the 0.3% and 0.5% lemongrass oil-treated leaves showed intact greenness on days 0–10, while yellow-green pigment and loss of moisture were observed on days 14–28 (Table 1). 

### 2.2. Dose-Dependent Reductions in P. fluorescens Treated with Oregano, Cinnamon, and Lemongrass Oil Microemulsions as Compared to Control 

**Figure 3:**
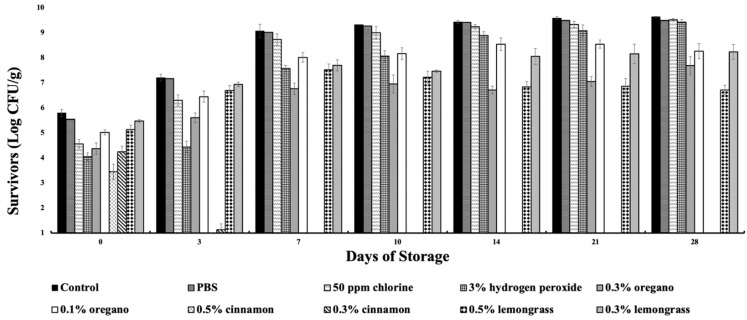
Survival of *P. fluorescens* on Iceberg lettuce treated with 0.3 and 0.1% oregano; 0.5 and 0.3% cinnamon; and 0.5 and 0.3% lemongrass oil microemulsions for 2 min and stored at 4 °C. All values are an average of three replicates. Error bars represent the standard deviation from the mean value.

Exposure of *P. fluorescens* to 0.5% and 0.3% cinnamon oil microemulsions showed that significant reductions were observed on day 0 that resulted in surviving populations of ca. 3.44-, and 4.24-log CFU/g, respectively, when compared to the positive control (*p* < 0.05) that had populations at 5.79-log CFU/g (Figure 3). Iceberg leaves treated with 0.5% and 0.3% cinnamon oil microemulsions showed biocidal activity against *P. fluorescens* population by days 3 and 7, respectively. The trend continued throughout the 28-day storage (*p* < 0.05). The 3% hydrogen peroxide treatment resulted in lower levels of surviving *P. fluorescens* populations (4.04–9.42 log CFU/g), in contrast with the 50 ppm chlorine and the PBS control that ranged in populations of 4.56–9.51 log CFU/g, and 5.54–9.49 log CFU/g, respectively, when compared to the positive control (5.79–9.63 log CFU/g) (*p* < 0.05). For the 0.3% oregano oil, lower surviving populations (4.37–7.68 log CFU/g) were observed during 28-day storage, when compared to the positive control (5.79–9.63 log CFU/g). The 0.5% lemongrass oil microemulsion demonstrated lower levels of *P. fluorescens* survivors (5.13–7.51 log CFU/g) throughout days 0–28 when compared to the positive control. The 0.3% lemongrass and 0.1% oregano oil microemulsions were able to maintain lower surviving populations of 5.47–8.23 log CFU/g and 5–8.54 log CFU/g, respectively, when compared to the positive control that ranged from 5.79 to 9.63 log CFU/g (*p* < 0.05). 

### 2.3. Visual Inspection of P. fluorescens Inoculated Iceberg Lettuce Leaves Treated with Essential Oil Microemulsions during 28 Days of Storage

**Figure 4:**
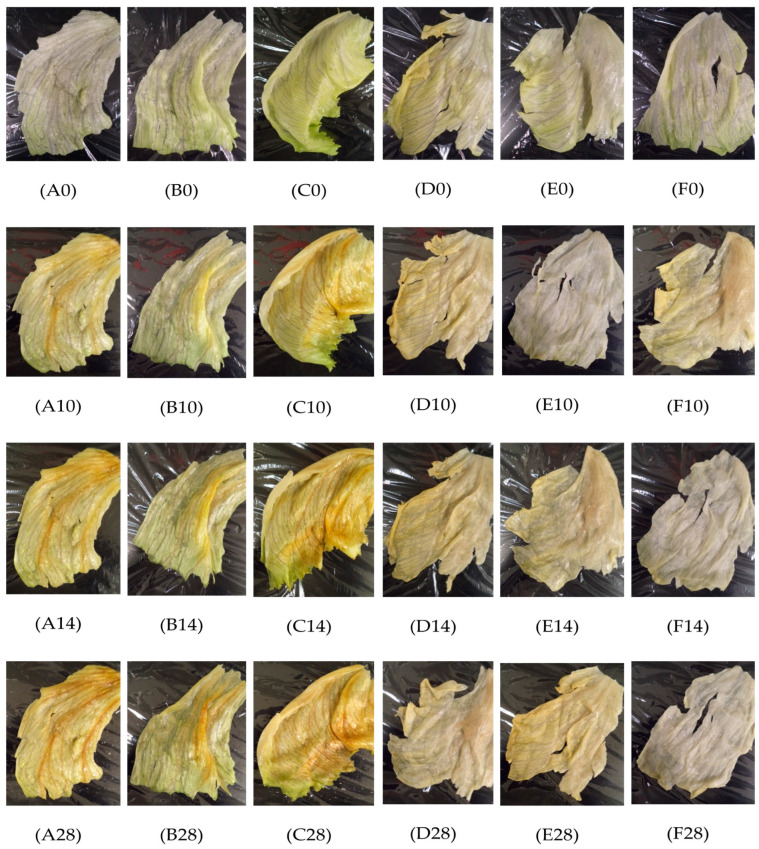
Visual observation of Iceberg lettuce leaves inoculated with *P. fluorescens*, and treated with 50 ppm chlorine, 3% hydrogen peroxide, and plant-based microemulsions and stored at 4 °C for 28 days. Letters denoted represent positive control (A0) on day 0, (A10) on day 10, (A14) on day 14, and (A28) on day 28; 50 ppm chlorine (B0) on day 0, (B10) on day 10, (B14) on day 14, and (B28) on day 28; 3% hydrogen peroxide (C0) on day 0, (C10) on day 10, (C14) on day 14, and (C28) on day 28; 0.5% cinnamon oil microemulsion (D0) on day 0, (D10) on day 10, (D14) on day 14, and (D28) on day 28; 0.3% cinnamon oil microemulsion (E0) on day 0, (E10) on day 10, (E14) on day 14, and (E28) on day 28; 0.3% oregano oil microemulsion (F0) on day 0, (F10) on day 10, (F14) on day 14, and (F28) on day 28.

The positive control Iceberg lettuce leaves remained visually appealing with visible signs of fresh, crisp texture and intact integrity on days 0–10; however, pinking and browning became apparent on days 10–28 (Figure 4). The PBS control leaves remained intact and visibly green throughout the storage period, except on days 21 and 28 where minimal browning was apparent (Table 2). The 50 ppm chlorine-treated leaves resulted in slight browning on day 3 and gradually increased in pinking and browning throughout the 28 days (Table 2). Hydrogen-peroxide-treated leaves showed slight browning at the apex of the leaves starting on day 7 and were continually browning up to day 28 (Table 2). For the leaves treated with a 0.3% oregano oil microemulsion, there was a visual discoloration on day 3 along with slight browning located at the cut edges of the leaves on days 7–28 (Table 2). Leaves treated with 0.1% oregano oil exhibited no discoloration on days 0–14, and at days 21 and 28 showed little to no signs of browning near the cut edges (Table 2). Both 0.3% and 0.5% cinnamon oil-treated leaves depicted slight browning, yellowish-green discoloration, with loss of moisture and turgidity on days 7–28. Both 0.3% and 0.5% lemongrass oil-treated leaves showed discoloration and wilting on day 7, and gradually increased loss of moisture and yellowing up to day 28 (Table 2). 

## 3. Discussion

### 3.1. Effectiveness of Plant-Based Microemulsion Treatments against E. coli O157:H7 and P. fluorescens on Iceberg Lettuce during 28-Day Storage

Microemulsions of both 0.3% oregano and 0.5% cinnamon oils effectively reduced the population of *E. coli* O157:H7 on Iceberg lettuce leaves during the 28-day storage (Figure 1). Reductions of *E. coli* O157:H7 were significantly higher than *P. fluorescens* for all antimicrobial treatments, including the chlorine and hydrogen peroxide treatments (Figure 3). The *E. coli* O157:H7 population was significantly reduced showing no detection of survivors on day 0 by 0.3% oregano and 0.5% cinnamon oils, making them the most effective microemulsion treatments. The second most effective treatments against *E. coli* O157:H7 were 0.3% cinnamon and 0.5% lemongrass oils showing no detectable survivors by day 3. On day 7, no *E. coli* O157:H7 survivors were recovered from the 0.1% oregano- and 0.3% lemongrass-treated leaves. All microemulsions showed no survivors by day 7, while the hydrogen peroxide and chlorine treatment had no survivors on days 21 and 28, respectively, which showed that the essential oil microemulsions were much more effective against *E. coli* O157:H7 than chlorine and hydrogen peroxide. In comparison, the most effective antimicrobials against *P. fluorescens* were both the 0.5% and 0.3% cinnamon oil microemulsions. The 0.5% and 0.3% cinnamon oil microemulsions exhibited bactericidal activity against *P. fluorescens* on days 3 and 7, respectively. Despite increased growth of *P. fluorescens* during the 28 days, variable reductions were observed in all the microemulsions that maintained lower surviving populations when compared to the positive control and the PBS, 50 ppm chlorine, and 3% hydrogen peroxide treatments. The trends in the reduction in *P. fluorescens* population continued throughout the storage time for all microemulsions, making 0.3% oregano oil more effective than 0.5% lemongrass, 0.3% lemongrass, and 0.1% oregano oil microemulsions. Increased resistance of the spoilage organism *P. fluorescens* to most of the natural microemulsions, except for the 0.3% and 0.5% cinnamon oil, was shown in this study and proves the cinnamon oil microemulsions to be effective against *P. fluorescens*. 

Gram-negative bacteria have an outer membrane that lies outside of a thin peptidoglycan layer that results in an increased resistance to essential oils. The external capsule of Gram-negative bacteria may limit or prevent the penetration of essential oils into the bacterial cells, whereas Gram-positive bacteria have cell walls that allow hydrophobic molecules to easily penetrate and act on both the cell wall and the cytoplasm, thus resulting in higher susceptibility to essential oils [18,22,23]. Holley and Patel [18] discussed pseudomonads and their increased resistance to antimicrobials such as linalool, carvacrol, thymol, and oregano. Pseudomonads are inherently abundant in causing food spoilage, so they have been considered as target organisms to be eliminated or inactivated in food products. Antimicrobial treatments of high concentrations have been reported to be effective [22]. In the present study, the most effective microemulsion treatment against both foodborne pathogen *E. coli* O157:H7 and spoilage organism *P. fluorescens* was 0.5% cinnamon oil, demonstrating complete bactericidal activity on days 0 and 3, respectively. The 0.3% cinnamon oil microemulsion showed no detection of survivors of *E. coli* O157:H7 and *P. fluorescens* on days 3 and 7, respectively. 

Furthermore, the rest of the microemulsion treatments such as oregano oil (0.3% and 0.1%) demonstrated no detection of survivors on days 0 and 7, respectively, for *E. coli* O157:H7, and resulted in reductions of 1.42–1.95 log CFU/g (for 0.3%) and 0.78–1.09 log CFU/g (for 0.1%) for *P. fluorescens* throughout the 28 days of storage. The 0.5% and 0.3% lemongrass microemulsions demonstrated no survivors by days 3 and 7, respectively, for *E. coli* O157:H7 and showed reductions of 0.66–2.12 log CFU/g (for 0.5%) and 0.32–1.4 log CFU/g (for 0.3%) for *P. fluorescens* during the 28 days. When comparing the efficacies of the antimicrobial microemulsions against both *E. coli* O157:H7 and *P. fluorescens*, the most effective treatments were 0.5% cinnamon oil, followed by 0.3% cinnamon, 0.3% oregano, 0.5% lemongrass, 0.3% lemongrass, and 0.1% oregano oils. 

### 3.2. A Comparison of the Efficacies of Essential Oil-Based Antimicrobial-PBS Suspensions and Essential Oil-Based Antimicrobial Microemulsions against E. coli O157:H7 and P. fluorescens 

The efficacy of edible films containing carvacrol, the active component of oregano oil, and cinnamaldehyde, the active component of cinnamon oil, against *E. coli* O157:H7 in bagged organic Iceberg lettuce salads has been investigated [24] and the findings of the present study are consistent with those of Zhu et al. [24], wherein carvacrol reduced the *E. coli* O157:H7 populations to below the detection limit (1 log CFU/g). Zhu et al. [24] also observed that 0.5% carvacrol, and 0.5% and 1.5% cinnamaldehyde present on any of the three types of edible films tested did not exhibit strong antibacterial activity. Results demonstrated they were not as effective as the 1.5% and 3% carvacrol, or 3% cinnamaldehyde [24]. In addition, various plant essential oils and active components were evaluated against *E. coli* and some of the most effective were oregano, thyme, cinnamon, clove bud, and lemongrass oils [25]. Gilling et al. [26] described the effects of essential oils against *E. coli* in vitro and showed significant reductions of 5.94-log CFU/mL by 0.3% lemongrass oil dissolved in PBS after a treatment time of 10 min when their initial inoculum was 7-log CFU/mL The present study’s findings indicate that on day 0, the 0.3% lemongrass microemulsion caused a 3-log CFU/g reduction in *E. coli* O157:H7 on Iceberg lettuce, when compared to the positive control that had a population of 5.86 log CFU/g. Significant reductions for both the 0.1% and 0.3% oregano and 0.3% and 0.5% cinnamon oil microemulsion treatments were observed in our study. Furthermore, this suggests the potential of natural antimicrobials to exhibit higher reductions in *E. coli* O157:H7 when solubility is improved in the essential oil components by means of an emulsifying agent. 

Only a few studies have evaluated the effects of plant-based antimicrobials on natural microflora, which could also include some spoilage microorganisms of fresh-cut vegetables. Yossa et al. [19] investigated the effects of cinnamaldehyde, clove, rosemary, and thyme oil formulations on native microbiota of Iceberg lettuce over 14 days of storage and found that the populations of both mesophilic and psychotropic bacteria increased significantly, resulting in ca. 7-log CFU/g. The present study showed similar findings, since the efficacy of most microemulsions against *P. fluorescens* was not different from the control except for the 0.3% and 0.5% cinnamon oil microemulsions. However, Yossa et al. [19] used Tween-20 to dissolve the essential oils, which suggests that Tween-20 may be ineffective in improving the efficacy of plant-based antimicrobials. Similarly, Moore et al. [27] studied the antimicrobial activities of olive extract, apple extract, and hibiscus concentrate against background microflora on organic Iceberg lettuce over storage period of 3 days. By day 3, reductions of 2.5, 0.9, and 1.0-log CFU/g were observed for 5% olive, 5% apple, and 30% hibiscus, respectively. The findings of Moore et al. [27] and the present study are consistent in suggesting that plant-based antimicrobials are effective against background microflora. Roller and Seedhar [28] evaluated the antimicrobial activities of carvacrol and cinnamic acid against spoilage microflora present in kiwifruit at concentrations below 20 mM (ca. 0.3%) during a storage period of 21 days at 4 °C. Their findings demonstrated that lower concentrations of 1 mM carvacrol and cinnamic acid prevented the appearance of visible spoilage on kiwifruit while inhibiting the growth of microflora for 5 days at 4 °C. For the higher concentrations of carvacrol and cinnamic acid treatments (10 and 15 mM), the antimicrobial activities reduced the microflora of fresh-cut kiwifruit throughout the 21 days of storage, with visual browning, and pungent unpleasant aroma from the fruit. The present study showed that both oregano and cinnamon oil microemulsions have the potential to be used against natural spoilage microflora on fresh-cut Iceberg lettuce without negatively impacting the organoleptic qualities at low concentrations. 

Even though the oregano and lemongrass oil microemulsions caused a limited reduction in *P. fluorescens* during the 28-day storage period, this may provide benefits since the indigenous microflora may serve as nearby competitors or have the potential to exhibit antagonistic activity against foodborne pathogens on fresh, minimally processed fruits and vegetables [11]. Babic et al. [29] mentioned that *P. fluorescens* biovars can produce siderophores that inhibit the growth of *Listeria monocytogenes* and demonstrated that the *P. fluorescens* biovar I had the strongest inhibitory effect against *L. monocytogenes* in mixed TSB cultures. This suggests that different biovars of the same species can have different antagonistic activities against *L. monocytogenes* due to the wide range of microbial competition [29]. The inhibitory properties exhibited by the potential dual combination of spoilage bacterial cultures such as *P. fluorescens* and natural microemulsions can serve as beneficial interactions that may be used to improve the safety of leafy greens. For example, Schuenzel and Harrison [11] discussed the inhibitory effects of the shredded lettuce native microbiota isolate *P. fluorescens* against multiple foodborne pathogens such as *E. coli* O157:H7, *L. monocytogenes, Salmonella* Montevideo, and *Staphylococcus aureus*. The benefits offered by these protective cultures should serve as a supplement to the existing handling and packaging practices for fresh-cut produce. 

### 3.3. Appearance and Visual Observation of Iceberg Lettuce Inoculated with E. coli O157:H7 and P. fluorescens, Treated with Plant-Based Microemulsions and Stored for 28 Days

Joshi et al. [30] evaluated the impact of plant-based antimicrobial washes on the sensory properties of organic Iceberg lettuce, and their results are consistent with the findings of the present study that oregano oil had the greatest impact on the color of Iceberg lettuce. The textural changes observed throughout our study suggest that those could be indicative of quality loss, since the non-uniformity in texture of Iceberg lettuce can render more susceptibility to physical damage [30]. Joshi et al. [30] also suggested the importance of incorporating an emulsion to improve the solubility and efficacy of essential oils when applied to food products, as this will also reduce the concentration needed to prevent negative impacts on the sensory attributes of the food matrix. 

It has been suggested that major qualities of minimally processed fruits and vegetables include color and appearance, flavor, texture, and nutritional value of the perishable commodity [31]. All of these play a significant role in the spoilage of leafy greens, since microbial spoilage is influenced by the presence of cut surfaces and high moisture levels. Furthermore, it has been demonstrated that the production of spoilage-related factors is due to the response of environmental signals that may include nutrient deprivation, pH changes, and temperature changes [6]. The visual observation of Iceberg lettuce leaves suggested that the higher concentration of the microemulsions caused light browning of leaves after 2 min treatment for some of the leaves, depending on the affected area of the leaf. Previous studies [28] have discussed that spoilage of fresh-cut kiwifruit was not visualized when treated with 1 mM carvacrol for 1 min throughout 5 days of storage at 4 °C, and our study demonstrated that discoloration was not present immediately after 2 min of 0.3% nor 0.1% oregano oil microemulsion treatments nor in the first 5 days of storage at 4 °C. 

Studies have elucidated that factors associated with lettuce spoilage are multifaceted. Lettuce spoilage can be due to the leaves being affected by the physical damage caused on their intact surface such that the edges can become bruised and discolored [32]. As seen in our study, the visual appeal of the Iceberg lettuce leaves inoculated with *E. coli* O157:H7 and treated with microemulsions was good during the 28 days of storage. For the Iceberg leaves inoculated with *P. fluorescens* and treated with microemulsions, minimal browning and discoloration were observed on day 7, and the visual appeal gradually decreased as storage time increased up to day 28. King et al. [6] suggested that the percentage of discolored leaves and spoilage microflora growth increased with longer storage time (26 days), during which the visual quality decreased during storage at 2 °C. Our findings are consistent with those of King et al. [6] since the *P. fluorescens* positive control leaves showed apparent signs of decay by day 14. Furthermore, this supports our findings that the microemulsions used in the current study can result in better visual appeal than untreated leaves inoculated with *P. fluorescens*.

Spoilage is attributed to factors such as lettuce metabolism, which can be controlled by pH changes and temperature fluctuations that can cause the lettuce tissue to face stressful environments and become susceptible to microbial invasion [8]. Visual indication of spoilage is best described as browning, or discoloration nearby cut surfaces, and/or loss of turgidity. Spoilage occurs by various mechanisms, which may include: (1) development in parallel with microbial growth, though not exclusively of microbial origin, and (2) plant metabolic actions caused by external factors such as biochemical, and physiological activities that occur in the vegetable tissue. The poor visual appeal observed on the positive control during days 21 and 28 may be due to the loss of turgidity, browning of the cut edges, discoloration, and “pinking” that is caused by polyphenol oxidase activity [18]. Moreover, the influence of nutrient release on the cut edges of the plants may induce increased growth of spoilage microflora, along with potential pathogens that rely on the vulnerability of the cell membrane within the plant tissue [10]. The integrity of fresh fruits and vegetables post-harvest has and remains a critically important factor that contributes to the shelf-life and consumer acceptability. For example, Barth et al. [32] have discussed that, upon harvest, fresh-cut produce usually benefits from rapid cooling to slow the product metabolism, and growth of spoilage microflora. Doing so will reduce the rate of metabolism that will also reduce product respiration, which results in the reduction in product deterioration [32]. In the present study, the leaves were unable to regain turgidity and remain intact. The apparent signs of wilt and decay during the 28-day storage period may have been caused by the plant’s metabolism and respiration activity. This possible effect could have resulted in the increased susceptibility of microemulsion-treated leaves to microbial attachment of *E. coli* O157:H7 and *P. fluorescens* via the wounded and cut edges of the leaf. Throughout the study, generally most of the microemulsion-treated leaves had a better visual appeal than untreated leaves despite the increased growth of *P. fluorescens*. 

### 3.4. Concentration and Storage Time-Dependent Reductions in E. coli O157:H7 and P. fluorescens Populations on Iceberg Lettuce during 28-Day Storage 

The results of the present study are consistent with those of Zhu et al. [24] in that the activities of the 0.3% and 0.5% cinnamon microemulsions against *P. fluorescens* were storage-time dependent during the 28 days evaluated. Other microemulsions such as oregano and lemongrass oil treatments responded variably. For example, the antimicrobial activity of 0.3% lemongrass was not time dependent due to decreased reductions in treatment efficacy on days 3,14, and 28 (Figure 3). 

## 4. Materials and Methods

### 4.1. Bacterial Culture Preparation and Media 

The pathogenic bacterial strain used was the *Escherichia coli* O157:H7 SEA13B88 dual antibiotic resistant (DABR) strain that was resistant to 100 µg/mL rifampicin and 100 µg/mL streptomycin. The spoilage bacterium used was *Pseudomonas fluorescens* DABR, resistant to 100 µg/mL tetracycline and 100 µg/mL streptomycin. Cultures for both microorganisms were maintained in cryovials at −80 °C and activated by transferring 100 µL in tryptic soy broth (TSB; Difco, Sparks, MD) that was incubated overnight (18–22 h) at 37 °C and 25 °C for *E. coli* O157:H7 and *P. fluorescens*, respectively. A fresh overnight culture (9- log CFU/mL) was prepared for each experiment and diluted using buffered peptone water (BPW; Difco) to a concentration of 6-log CFU/mL for use in experiments. 

### 4.2. Fresh Produce and Antimicrobials Used

The heads of conventionally grown Iceberg lettuce were obtained from local grocery stores in Tucson, Arizona. The oregano and cinnamon oils (made from 100% pure *Origanum vulgare* and *Cinnamomum cassia*, respectively) were obtained from the Lhasa Karnak Company (Berkeley, CA, USA). Oregano oil consists of carvacrol, β-fenchyl alcohol, thymol, and γ-terpinene [33]. The major constituents of cinnamon oil are (E)-cinnamaldehyde, linalool, β-caryophyllene, eucalyptol, and eugenol [34]. The lemongrass oil made from 100% pure *Cymbopogon flexuosus* leaves was obtained from NOW Foods (Bloomingdale, Illinois). The chemical composition of lemongrass oil are neral, citral, and geranyl acetate [35].

### 4.3. Preparation of Antimicrobial Microemulsions 

Dip solutions of lemongrass at 0.3 and 0.5% (*v/v*), cinnamon at 0.3 and 0.5% (*v/v*), and oregano oils at 0.1 and 0.3% (*v/v*) concentrations were prepared in sterile deionized water. A plant-based emulsifier (Quillaja saponin; Sigma-Aldrich, St. Louis, MO) at 0.0001% concentration was added to each of the treatment solutions. The mixtures were both manually shaken, and stomached (Seward, London, UK) at normal speed (230 paddle speed/min) to vigorously mix the microemulsion treatments for 1 min immediately before use. Dip solutions of phosphate-buffered saline (PBS, pH 7), 50 ppm sodium hypochlorite with pH adjusted to 6.5 with citric acid, and 3% hydrogen peroxide were also prepared. Treatment dip solutions were used immediately after preparation.

### 4.4. Inactivation of E. coli O157:H7 and P. fluorescens on Iceberg Lettuce by Essential Oil Microemulsions 

The outer four leaves from the iceberg lettuce head were removed and discarded. Whole-leaf samples of Iceberg lettuce were weighed (10 g ± 0.5 g each). To reduce background microflora, the leaves were washed with 3% sodium hypochlorite for 5 min, then leaves were washed thoroughly under sterile deionized water for 1 min for a total of 3 times. Samples were placed under UV light (254 nm) in a biohood for 30 min with each side of the leaf exposed for 15 min [27]. The samples were then dip inoculated for 2 min in a buffered peptone water (BPW) solution (6-log CFU/mL) of *E.coli* O157:H7 or *P. fluorescens* and dried for 1 h under the biohood to allow bacterial attachment to the leafy greens. Control samples were taken after inoculation and after 1 h of drying for the determination of inoculation levels present on the leaf surface [27]. Each leaf sample was submerged in one of the antimicrobial treatment solutions (200 mL) for 2 min with manual gentle agitation and in PBS without antimicrobials. After treatment, the samples were placed in individual stomacher bags, sealed and incubated at 4 °C. Samples were taken on days 0, 3, 7, 10, 14, 21, and 28 for the enumeration of surviving *E. coli* O157:H7 and *P. fluorescens*. Leaf samples (10 g) were stomached in Dey–Engley (D/E) neutralizing broth (Hardy Diagnostics, Springboro, OH) for 1 min, and enumeration of survivors following treatment was performed. Samples were serially diluted in 0.1% peptone water and spread-plated on sorbitol MacConkey agar with 100 µg/mL of rifampicin and 100 µg/mL of streptomycin (Hardy Diagnostics, Springboro, OH), and *Pseudomonas* agar F with 100 µg/mL of tetracycline and 100 µg/mL of streptomycin (Difco, Becton Dickinson), for enumerating the surviving populations of *E. coli* O157:H7 and *P. fluorescens*, respectively. Visual inspection of lettuce leaves for any signs of wilting, decay, or discoloration, etc. was done and observations were photographed on days 0, 3, 7, 10, 14, 21, and 28. Colonies were counted after 48 h incubation for *E. coli* O157:H7 at 35 °C and 24 h incubation for *P. fluorescens* at 23 °C. All experiments were repeated three times. 

### 4.5. Statistical Analyses 

Colony counts of *E. coli* O157:H7 obtained from duplicate sorbitol MacConkey agar plates and colony counts of *P. fluorescens* enumerated from duplicate Pseudomonas F agar at each sampling period were converted to log CFU/g. Each experiment was repeated three times per treatment. Data were analyzed by a one-way ANOVA with TUKEY statement for pair-wise comparison (Minitab 19 Software; Minitab Inc., State College, PA) for interaction effects of oregano, cinnamon, and lemongrass oil concentrations. Significant differences are defined at *p* < 0.05.

## 5. Conclusions

The present study demonstrated that the effects of the plant-based microemulsion treatments are concentration and storage time-dependent against two antibiotic-resistant organisms on Iceberg lettuce: foodborne pathogen *E. coli* O157:H7 and spoilage microorganism *P. fluorescens*. This study demonstrated that alternative approaches to chlorine-based sanitation, such as the potential applications of plant-based antimicrobial microemulsions, should be sought out by the fresh produce industry for the prevention of resistant foodborne pathogens as well as spoilage microorganisms that are associated with leafy greens. For both *E. coli* O157:H7 and *P. fluorescens*, the microemulsion treatments not only provided bactericidal and/or inhibitory effects during the 28-day storage period, but some of the microemulsion treatments also resulted in better visual appeal throughout the 28 days of storage when compared to the untreated positive control, PBS-, 50 ppm chlorine-, and 3% hydrogen peroxide-treated leaves. Regarding the safety of Iceberg lettuce from *E. coli* O157:H7, the most effective microemulsions were 0.3% oregano and 0.5% cinnamon. However, the most effective microemulsions for the quality of Iceberg lettuce regarding *E. coli* O157:H7 were 0.3% lemongrass and 0.1% oregano. For *P. fluorescens*, the most effective microemulsion for the quality of Iceberg lettuce was 0.1% oregano. These significant findings suggest that further studies are needed in seeking a microemulsion that can both serve as an effective biocidal agent against foodborne pathogens and demonstrate improved quality of the perishable commodity in the presence of natural spoilage microflora. There is potential for combination treatments to inactivate pathogens on minimally processed leafy greens and/or extend the shelf-life of leafy greens by delaying the growth of spoilage organisms. The antagonistic effects of spoilage microflora such as *P. fluorescens* when combined with microemulsions against foodborne pathogens also merit further studies. Additional research is needed to determine the effectiveness of these plant-based microemulsions against other foodborne pathogens in other fresh-cut leafy greens, as well as in other types of food matrices, in addition to an assessment of the sensory attributes of treated fresh-cut leafy greens during long-term storage, to determine consumer acceptability. The present study complements and extends our previous reports on the inactivation of pathogenic bacteria by plant-based antimicrobials on contaminated conventional and organic celery, Iceberg lettuce, Romaine lettuce, and spinach and on the sensory properties of antimicrobial treated leafy greens [30,36,37,38,39,40,41,42].

## Figures and Tables

**Table 1:** Visual observation results of Iceberg lettuce inoculated with *E. coli* O157:H7, treated with plant-based microemulsions, 50 ppm chlorine, and 3% hydrogen peroxide and stored for 28 days at 4 °C.

	D0	D3	D7	D10	D14	D21	D28
**Controls**							
Positive	NA	NA	BR	BR	BR	BR	BR, W
Negative	NA	NA	NA	NA	NA	NA	W
PBS	NA	NA	NA	W	NA	BR, LG	BR, W
**Treatments**							
50 ppm chlorine	NA	NA	NA	NA	BR, LM	BR, LM	BR, W
3% H_2_O_2_	NA	NA	BR	BR	BR	BR, W	BR, W
0.3% oregano	NA	NA	NA	LG, LM	LG, LM	LG, LM	LG, LM
0.1% oregano	NA	W	W	W	W	W	BR, W
0.5% cinnamon	NA	LG	LG	LG	LG	LG, LM	BR, LG
0.3% cinnamon	NA	DSI	W	W, LG	W	W, LG	BR, W, LG
0.5% lemongrass	NA	NA	NA	NA	NA	NA	W
0.3% lemongrass	NA	NA	NA	W	W, LG, LM	LM	W, LM

NA—normal appearance; BR—browning; W—wilt; D—decay; DSI—disintegration; LG—loss of green; LM—loss of moisture.

**Table 2:** Visual observation results of Iceberg lettuce inoculated with *P. fluorescens*, treated with plant-based microemulsions, 50 ppm chlorine, and 3% hydrogen peroxide and stored for 28 days at 4 °C.

	D0	D3	D7	D10	D14	D21	D28
**Controls**							
Positive	NA	NA	BR	BR	BR	BR	BR, SM
Negative	NA	NA	NA	NA	NA	SM	W, LG, SM
PBS	NA	NA	W	W	W	BR, W, SM	BR, W, SM
**Treatments**							
50 ppm chlorine	NA	NA	BR	BR	BR	BR, W, LM	BR, W, LM
3% H_2_O_2_	NA	NA	NA	BR, SM	BR, D, SM	BR, D, SM	BR, D, SM
0.3% oregano	NA	LM	LM	LM	LM	LM	LM
0.1% oregano	NA	NA	NA	NA	NA	NA	W
0.5% cinnamon	NA	NA	NA	NA	W	LM, SM	LM, SM
0.3% cinnamon	NA	NA	NA	NA	W	LM, SM	LM, SM
0.5% lemongrass	NA	NA	LG, LM	LG, LM	W, LG, LM	W, LG, LM	W, LG, LM
0.3% lemongrass	NA	NA	W, LG	NA	W, LG	W, LG	W, LG

NA—normal appearance; BR—browning; W—wilt; D—decay; DSI—disintegration; LG—loss of green; LM—loss of moisture; SM—slime appearance.

## Data Availability

Not applicable.
